# Conserved alternative and antisense transcripts at the programmed cell death 2 locus

**DOI:** 10.1186/1471-2164-8-20

**Published:** 2007-01-18

**Authors:** Ondrej Mihola, Jiri Forejt, Zdenek Trachtulec

**Affiliations:** 1 Institute of Molecular Genetics, Academy of Sciences of the Czech Republic, Center for Applied Genomics, Videnska 1083, 142 20 Prague, Czech Republic

## Abstract

**Background:**

The programmed cell death 2 (*Pdcd2*) gene on mouse chromosome 17 was evaluated as a member of a highly conserved synteny, a candidate for an imprinted locus, and a candidate for the Hybrid sterility 1 (*Hst1*) gene.

**Results:**

New mouse transcripts were identified at this locus: an alternative *Pdcd2 *mRNA skipping the last two coding exons and two classes of antisense RNAs. One class of the antisense RNA overlaps the alternative exon and the other the entire *Pdcd2 *gene. The antisense RNAs are alternative transcripts of the neighboring TATA-binding protein gene (*Tbp*) that are located mainly in the cell nucleus. Analogous alternative PDCD2 forms truncating the C-terminal domain were also detected in human and chicken. Alternative transcripts of the chicken *PDCD2 *and *TBP *genes also overlap. No correlation in the transcription of the alternative and overlapping mRNAs was detected. Allelic sequencing and transcription studies did not reveal any support for the candidacy of *Pdcd2 *for *Hst1*. No correlated expression of *Pdcd2 *with the other two genes of the highly conserved synteny was observed. *Pdcd2*, *Chd1*, and four other genes from this region were not imprinted in the embryo.

**Conclusion:**

The conservation of alternative transcription of the *Pdcd2 *gene in mouse, human and chicken suggests the biological importance of such truncated protein. The biological function of the alternative *PDCD2 *is likely to be opposite to that of the constitutive form. The ratio of the constitutive and alternative *Pdcd2 *mRNAs differs in the tissues, suggesting a developmental role.

The identified *Tbp-*alternative *Pdcd2*-antisense transcripts may interfere with the transcription of the *Pdcd2 *gene, as they are transcribed at a comparable level. The conservation of the *Pdcd2*/*Tbp *sense-antisense overlap in the mouse and chicken points out its biological relevance. Our results also suggest that some cDNAs in databases labeled as noncoding are incomplete alternative cDNAs of neighboring protein-coding genes.

## Background

The mouse *Pdcd2 *gene has been mapped to mouse chromosome (Chr) 17, tail-to-tail to the gene for TATA-binding protein (*Tbp*), which makes it a candidate for the mouse *Hst1 *gene [1,2]. The distance between the stop codons of *Tbp *and *Pdcd2 *is 4.8 kb. The arrangement of these two genes is also conserved in human [1] and at least five nonmammalian vertebrates [3]. The proteasome subunit C5 gene (*Psmb1*) is syntenic with these two genes in the mouse, being located head-to-head with *Tbp*, and in all vertebrates investigated thus far [3]. These genes are nonrandomly distributed also in nonvertebrate genomes [1,4]. The coordinated expression of these three genes could be one of the possible reasons for this observation.

In the rat, transcription of the *Pdcd2 *(also called *Rp8*) gene was induced when thymocytes underwent programmed cell death caused by applying radiation or dexamethasone [5] and in fibroblasts after exposure to neutron radiation [6]. The amount of the rat protein increased in nuclei of kidney fibroblasts after prolonged hypoxia [7]. The mRNA of the human orthologous gene, *PDCD2*, was upregulated in peripheral blood mononuclear cells from patients with chronic fatigue syndrome [8]. However, the upregulation of the mouse and human orthologous genes has not been universally associated with apoptosis [9-11].

The product of the *Pdcd2 *gene and its orthologs consists of two domains: a MYND zinc-finger DNA-binding domain found in transcription regulators and a highly conserved C-terminal (CT) domain of unknown function. The human *PDCD2 *gene has two forms. The alternative, less abundant *PDCD2 *mRNA is formed due to a polyadenylation (polyA) signal in the third intron. The resulting product thus carries the MYND, but not the CT domain [GenBank: NM_144781]. *PDCD2 *is negatively regulated by the B-cell lymphoma 6 (*BCL6*) gene in lymph node germinal centers of human tonsils [12], but no such repression was found in immortalized primary B cells [13]. *PDCD2 *is also a regulator of the host cell factor C1 (*HCFC1*) gene; mutations in *HCFC1 *arrest cells in the middle of the G1 phase of the cell cycle [14]. *PDCD2 *is a candidate for a tumor-suppressor gene on human Chr 6q27 [12-15]. *PDCD2 *and *TBP *are candidate type 1 diabetes susceptibility loci [16,17]. A possible haploinsufficiency effect at the 6q27 tumor-suppressor locus has been suggested [15]. A haploinsufficiency gene (*Thl1*) is also located on mouse Chr 17, in a region that includes *Pdcd2 *[18-20]. The mouse *Pdcd2 *gene has been considered a candidate imprinted gene, as it is differentially expressed in 9.5-day-post-coitum (E9.5) parthenogenetic versus androgenetic mouse embryos of the C57BL/6J (henceforth B6) strain [21].

The aim of this study was to characterize in detail transcription within the *Pdcd2 *locus and its vicinity, as well as its imprinting status and candidacy for the *Hst1 *locus. To elucidate the reasons for the conservation of the synteny surrounding *Pdcd2*, we also analyzed the transcription and imprinting of the neighboring genes.

## Results

### Alternative transcript of the mouse *Pdcd2 *gene

**Figure 1:**
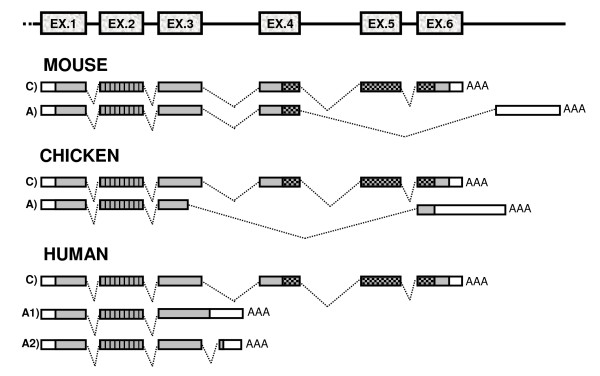
**Schematic representation of the constitutive (C) and alternative (A) transcripts encoding PDCD2 in the mouse, chicken and human**. Empty and grey rectangles represent the untranslated regions and ORFs, respectively. Vertically hatched rectangles represent the MYND zinc-finger domain in exon 2, and crosshatched rectangles represent the conserved domain of unknown function (CT) coded by exons 4, 5, and 6. EX., exon; AAA, polyA tail.

To look for isoforms of the mouse *Pdcd2 *mRNA, we used the BLAST program to identify ESTs matching the *Pdcd2 *genomic sequence. The presence of a putative alternative *Pdcd2 *exon, which starts 1217 bp from the 3' end of the constitutive *Pdcd2 *transcript, was detected [GenBank: AA199080, clone 657319 from E12.5 embryo]. The EST clone was then sequenced from its 3' end and a polyA tail was found. The 3' end was preceded by a polyA signal, AATAAA. No A-rich sequence that could cause a spurious priming of the oligo-dT primer during cDNA preparation was found in the genomic DNA. The alternative transcript of *Pdcd2 *was confirmed by RT-PCR of testicular RNA (data not shown). The alternative mRNA contained a new exon on its 3' end, which replaced the fifth and sixth constitutive *Pdcd2 *exons and thus also changed the open reading frame (ORF). The alternative transcript thus does not contain the highly conserved CT domain, as with the human *PDCD2 *isoform 2 (Fig. 1). However, unlike the *Pdcd2 *ORF, the human ORF is truncated by the end of exon 3 [GenBank: NM_144781]. The 3' RACE method confirmed the 3' end of the alternative *Pdcd2 *transcript also in testicular RNA. The sequenced RACE product was the same as deduced from the EST clone 657319. The length of the *Pdcd2 *alternative exon was 1147 bp. The 3' end of the alternative exon matched another EST sequence from E12 embryo (BB097798), but the alternative exon was 1156 bp in length according to this EST [GenBank: DQ906042].

### RNAs transcribed from the opposite strand of the *Pdcd2 *locus and its vicinity

**Figure 2:**
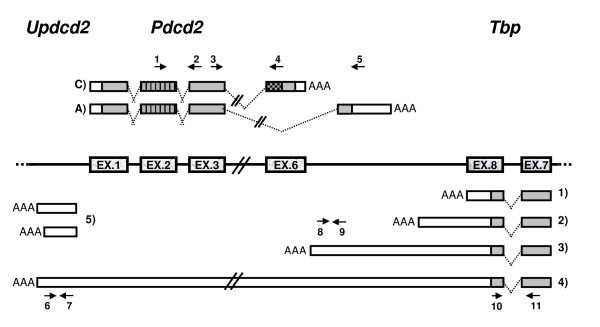
**Schematic representation of *Pdcd2 *and *Tbp *transcripts in mouse testes**. 1) to 4): forms of *Tbp *transcripts; 1) constitutive *Tbp *transcript, 2) alternative *Tbp *transcript not reaching *Pdcd2*, 3) *Pdcd2as1 *transcript overlapping the entire alternative *Pdcd2 *exon, 4) *Pdcd2as2 *transcript, the longest *Tbp *alternative, overlapping the entire *Pdcd2 *gene, 5)*Updcd2 *– two putative transcripts upstream of *Pdcd2 *using the same promoter as *Pdcd2*. Numbered arrows represent the primers used for real-time PCR. Primer pair 1–2 detects both *Pdcd2 *transcripts, 3–4 just constitutive *Pdcd2*, 3–5 just alternative *Pdcd2*, 6–7 *Pdcd2as2*, 8–9 both antisense mRNAs (*Pdcd2as1 *and *Pdcd2as2*), 10–11 all forms of *Tbp*. For other legend see Fig. 1.

The BLAST output of the genomic region covered by the alternative *Pdcd2 *transcript also contained five other ESTs (AA792289 from skin, CA887845 from differentiated neural stem cells, AA792289 from skin, BY723771 from hypothalamus, and CX219724 from neurosphere). Due to their orientation, these ESTs could represent an antisense transcript of the alternative exon of *Pdcd2*. The opposite end of the AA792289 EST clone was therefore sequenced. The antisense orientation was confirmed by the presence of polyA tail preceded by a polyA signal (AATAAA). There was no A-rich sequence in the genomic DNA that would cause a mispriming of the oligo-dT primer during cDNA preparation. The 3' RACE method and sequencing were then used to identify the antisense transcript in mouse testis as well. The EST and RACE sequences were identical and matched the genomic *Pdcd2 *sequence in the entire alternative exon and a part of the alternative intron, 382 bp apart from the constitutive polyA site of *Pdcd2*. The sequences did not contain the sequence of the *Pdcd2 *constitutive exons (Fig. 2). By RT-PCR and sequencing, it was confirmed that this transcript (termed *Pdcd2as1*) is a novel alternatively polyadenylated form of the neighboring *Tbp *gene.

Four other ESTs, two from mammary tumor (AW211687 and AW212179) and two from a forelimb of E13 embryo (CJ049248 and CR514396) were identified upstream of the 5' end of *Pdcd2*, suggesting there may be a new gene transcribed from the same promoter as *Pdcd2*, but in the opposite orientation. This 5' region is not conserved in human. The transcription status of this mouse region was investigated by RT-PCR and RACE methods. A transcript encompassing this sequence upstream of the *Pdcd2 *gene was found in mouse testes and other tissues. At least two alternative forms of this transcript (termed *Updcd2*) formed by different polyadenylation were identified by 3' RACE and RT-PCR, one of them having the same 3'-end as the EST CR514396 (Fig. 2). 5' RACE detected the 5' end consistent with EST CJ049248 [GenBank: DQ906044]. The bi-directional function of the *Pdcd2 *promoter is also supported by our finding of two tags in the CAGE (Cap Analysis of Gene Expression) database [22]. The CAGE method measures the expression levels of transcription starting sites by sequencing 5' ends of transcripts prepared through modifying their caps. The CAGE tags T17F00DAE0F6 and T17F00DAE115 suggest that transcription also occurs in the *Updcd2 *direction. However, there are about ten times more CAGE tags in the *Pdcd2 *direction, suggesting the promoter activity is much stronger in the direction of *Pdcd2 *than in that of *Updcd2*.

On the other hand, these identified RNAs could also represent a novel alternative transcript of the *Tbp *gene, an antisense read-through of the entire *Pdcd2 *gene. Therefore, RT-PCR with a *Updcd2*-specific primer used for reverse transcription and PCR with primer pairs along the *Pdcd2 *and *Tbp *genes were used to identify this read-through transcript (henceforth *Pdcd2as2*). As the PCR products along the entire *Pdcd2 *gene, the *Pdcd2-Tbp *intergenic region and the last exons of *Tbp *genes were successfully amplified and sequenced, the *Pdcd2as2 *transcript appears to be another new alternative form of *Tbp *originating by alternative polyadenylation and encompassing the entire *Pdcd2 *gene in the antisense orientation [GenBank: DQ906043]. Thus, our data indicate that there is both a new alternative *Tbp *mRNA and a new gene sharing the *Pdcd2 *promoter.

Furthermore, the BLAST search of the *Tbp-Pdcd2 *intergenic region revealed nine ESTs, which apparently represent another alternative transcript(s) of the *Tbp *gene. Three 3' EST sequences (AU020825, AU019062, and the polyA-containing BG069472, all from the embryo) extend the previously characterized 3'UTR of *Tbp *by 416 bp. Six EST sequences (BQ934602 otocysts, BM945950 E18.5 brain, BG975205 mammary tumor, CK634594 E9.5-10.5 head, CB841791 E15.5 eye and BB068539 from E15 testes) overlap the former three ESTs and each other and extend by 1660 bp in the 3'direction. A 3'RACE product was obtained from a testicular RNA and sequenced. It contains the polyadenylation signal ATTAAA and carries the same end as EST BB068539. No A-rich sequence that could cause an oligo-dT mispriming during cDNA preparation was found in the genomic DNA.

Taken together, besides the *Updcd2 *transcripts, there are three to four alternative *Tbp *forms transcribed from the opposite DNA strand in the *Pdcd*2 locus and its vinicity, including *Pdcd2as1*, *Pdcd2as2*, 3'RACE-confirmed mRNA, and the transcript suggested by the three embryonic ESTs incl. BG069472. The alternative mRNAs of the *Tbp *gene arise by alternative polyadenylation and differ in overlaps with the *Pdcd2 *gene (Fig. 2).

### Transcription analyses of mouse *Pdcd2 *and Tbp loci

**Figure 3:**
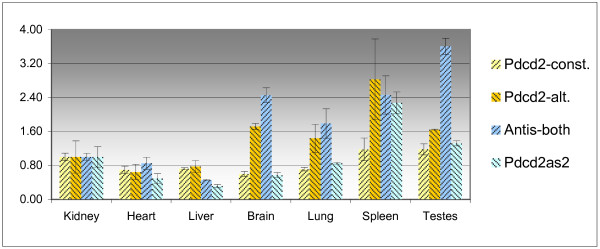
**Quantification of the expression of *Pdcd2 *and *Tbp *transcripts in seven mouse tissues**. For better visualization of the results, the relative quantity in the kidney was taken as one; const., constitutive transcript; alt., alternative transcript, Antis-both, *Pdcd2as1 *and *Pdcd2as2 *transcripts.

**Figure 4:**
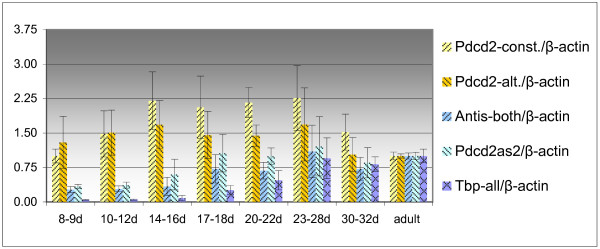
**Relative quantification of the expression of *Pdcd2 *and *Tbp *transcripts in the testes of mice of different age; **normalized by the expression of β-actin. Adult mice were 2 to 2.5 months old. For better visualization of the results, the relative quantity in the adult mouse testes was taken as one; for other legend see Fig. 3.

The expression of all identified *Pdcd2 *and *Tbp *transcripts was detected by RT-PCR in all eight tissues examined, as well as in different developmental stages of the testis (data not shown). To find whether the antisense transcripts could participate in the regulation of the ratio between the mRNA isoforms [23,24], the level of expression of *Pdcd2 *and *Tbp *alternative transcripts was more accurately determined by quantitative real-time RT-PCR (QRT-PCR) in seven mouse tissues (Fig. 3), as well as in developmental stages of the testis (Fig. 4) using primers detecting the *Pdcd2 *constitutive, alternative, *Pdcd2as1 *and *Pdcd2as2 *transcripts (primers Fig. 2, Additional file 1).

In tissues, relatively higher levels of the alternative *Pdcd2 *transcript correlated with upregulation of the *Pdcd2-*antisense alternative *Tbp *transcripts. However, no such relationship was detected in the developmental stages of testes (Fig. 4).

**Figure 5:**
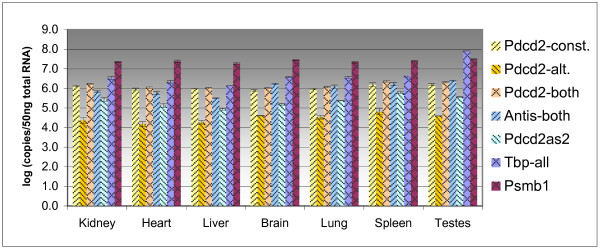
**Absolute quantification of the expression of *Pdcd2*, *Tbp *and *Psmb1 *transcripts in seven mouse tissues**. The values represent the common logarithm of the number of copies of the particular transcript in the sample corresponding to the amount of 50 ng of total RNA.

The levels of all examined transcripts were determined using the absolute quantification method in seven mouse tissues (Fig. 5, Table 1). The levels of the alternative *Pdcd2 *transcripts are on average 40 times lower compared to the level of the constitutive *Pdcd2 *transcript in the analyzed tissues. The *Pdcd2as2 *transcript was expressed in tissues on average 5 times less than the constitutive *Pdcd2*. The levels of the *Pdcd2as1 *transcript in tissues were comparable to that of the constitutive *Pdcd2 *transcript. The level of all *Tbp *forms (detected by primers from the TBP-coding region) in the testes was nearly 30 times higher than the level of *Pdcd2*-antisense alternative *Tbp *transcripts, in contrast to only 3-fold difference in other tissues (Table 1).

**Figure 6:**
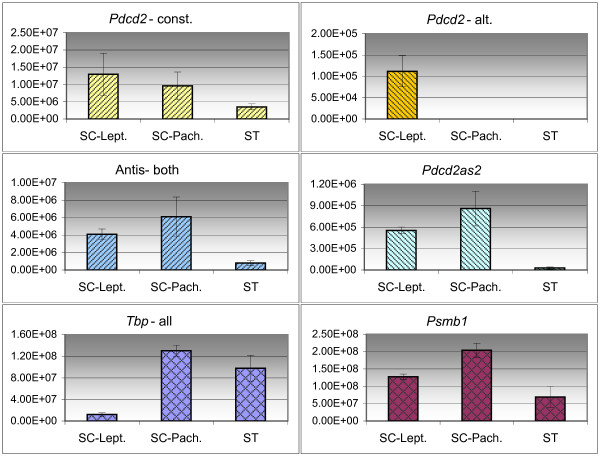
**Absolute quantification of the expression of *Pdcd2 *and *Tbp *transcripts in flow-sorted spermatogenic cell populations**. The values on axis x represent the number of copies of the particular transcript in the sample corresponding to the amount of 50 ng of total RNA. SC-Lept, leptotene+zygotene spermatocytes; SC-Pach., pachytene spermatocytes; ST, spermatids; const., constitutive transcript; alt., alternative transcript; Antis-both, *Pdcd2as1 *and *Pdcd2as2 *transcripts.

The variations of the expression of the sense/antisense (SA) transcripts in different cell types could make the SA relationship in developmental stages of the testis undetectable. To identify the *Pdcd2*/*Tbp *SA transcript ratios in homogenous cell populations, pure flow sorted populations of spermatogenic cells were isolated and the mRNA levels of *Pdcd2 *and antisense *Tbp *transcripts were assessed by QRT-PCR (Fig. 6). The alternative *Pdcd2 *transcript was detected just in the population of leptotene spermatocytes. The expression of the antisense transcripts exhibited neither positive nor negative correlation with this restricted alternative *Pdcd2 *expression, suggesting that there is no regulation of the *Pdcd2 *alternatives by the antisense RNAs in these cells (Fig. 6). Also, the higher quantity of total *Tbp *transcripts was detected in pachytene spermatocytes and spermatids.

**Figure 7:**
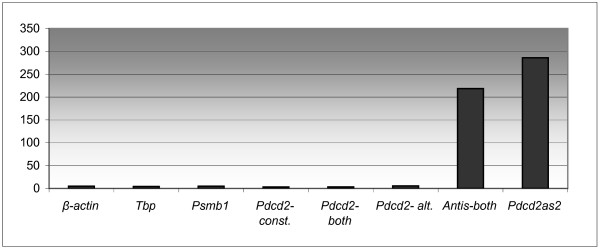
**The ratio of transcripts in nuclear versus cytoplasmic RNA fractions in adult B6 testes**. const., constitutive transcript; alt., alternative transcript, Antis-both, *Pdcd2as1 *and *Pdcd2as2 *transcripts.

Long perfect dsRNA duplexes, resulting from the antisense transcription, can be substrates for adenosine deaminase (ADAR) enzymes that catalyze the hydrolytic deamination of adenosines to inosines. Massive A/I editing can cause the subsequent retention of the edited RNAs in the nucleus [25,26]. To test whether the *Pdcd2as1 *or *Pdcd2as2 *transcripts can drive the expression of the sense *Pdcd2 *transcripts by such a mechanism, the mRNA levels of *Pdcd2 *and both their antisense RNAs were measured in cytoplasmic and nuclear fractions by QRT-PCR. The nuclear/cytoplasmic ratios of the tested genes disclose the nuclear localization of *Pdcd2as1 *and *Pdcd2as2 *transcripts, but cytoplasmic localization of the *Pdcd2 *mRNAs (Fig. 7).

### Alternative and antisense transcription at the chicken, rat, and human *PDCD2 *loci

Recently, the chicken region containing the *PDCD2*, *TBP*, and *PSMB1 *genes was sequenced and major transcripts identified by RT-PCR ([3], [GenBank: AY376311]). The genes are in the same order and orientation as their mouse and human orthologs. The organization of the coding exons of the chicken *PDCD2 *gene (also called Q6JLB0_CHICK) is the same as in the mouse and human. Provided that the alternative and antisense transcripts are important for the function of the *Pdcd2 *orthologous genes, these features should also be conserved in the chicken genome. Conserved overlapping transcription could also shed light on the synteny conservation. We therefore looked for alternative and antisense transcription at the chicken *PDCD2 *locus by EST analysis, RACE and RT-PCR. A putative alternative *PDCD2 *transcript that suggests skipping exons 5 and 4 and a part of exon 3 was detected in the dbEST database (BU309667 from the heart, CO773817 from the testes). The presence of the alternative transcript in chicken was confirmed by RT-PCR and sequencing of testicular RNA. This alternative mRNA does not contain the CT domain, as with the mouse and human alternative transcripts. However, unlike mouse or human, the chicken ORF is truncated before the end of exon 3 (Fig. 1, [GenBank: DQ906045]).

The overlapping transcription of chicken *PDCD2 *and *TBP *was suggested by ESTs (BU272933 from cerebrum, BU462305 and BU476463 from the ovary) and it was confirmed by RT-PCR of testicular RNA using primers from the intergenic region in combination with primers from the exons of *TBP *and *PDCD2*, respectively. The sequence of the PCR products indicated an overlap of at least 80 bp between alternative *PDCD2 *and *TBP *transcripts [GenBank: DQ906046, DQ906047]. The SA overlap is thus conserved in chicken. Our RT-PCR data also indicate that the chicken *Tbp *gene has another alternatively polyadenylated form even longer than the one published by Yamauchi et al. [27].

The human *PDCD2 *has an alternative transcript formed by polyadenylation in the third intron [GenBank: NM_144781]. However, EST BQ187150 from fetal eye and eight others suggest alternative splicing rather than alternative polyadenylation (Fig. 1, human transcript 2A). No overlapping transcription in the human is indicated by the ESTs, although there are 18 ESTs (including polyadenylated AW207335) in the *PDCD2-TBP *intergenic region, which could represent an alternative human *PDCD2 *isoform(s), generated by alternative polyadenylation. However, the analysis of this region is hindered by an A-rich sequence.

There is also a putative alternatively polyadenylated transcript of *Rp8*, the rat homolog of the mouse *Pdcd2 *gene (polyadenylated rat EST AI704628 from E16.5 ventricle). Two ESTs matching the *Rp8*-*Tbp *intergenic region (AI412868 from the brain and CO571404 from the testes) suggest overlapping transcription in rat, because they are in the opposite orientation. There are also two ESTs in the third *Rp8 *intron (CB730473 and CB727152, hypothalamus) in the opposite direction than the *Rp8 *gene. The alternative form of the *Rp8 *gene lacking the CT domain has not been suggested by any EST yet.

### Analysis of coordinated expression of three genes of a highly conserved synteny

**Figure 8:**
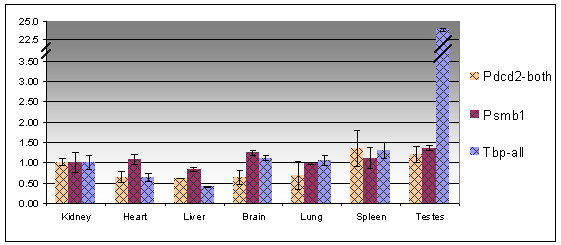
**Quantification of the expression of *Pdcd2*, *Tbp *and *Psmb1 *transcripts in different mouse tissues**. For better visualization of the results, the relative quantity in kidney was taken as one.

The orthologs of *Pdcd2 *and its neighboring genes *Tbp *and *Psmb1 *are nonrandomly distributed in eukaryotic genomes [1,3], but the reason for this observation is unknown. As one of the possible reasons could be the coordinated expression of these three genes, we have tested this hypothesis by QRT-PCR in mouse tissues. The three genes are housekeeping, but the expression of *Tbp *has been reported to be highly elevated in the testis [28], while the other genes were not tested in this organ. As shown in Fig. 8, *Tbp *but not *Pdcd2 *or *Psmb1 *were overexpressed in the testis. Thus, there are some regulatory elements of *Tbp *not accessible by the other two genes. Indeed, analyses of a much broader panel of tissues and stages by microarrays suggested a different pattern of expression for these three genes [29]. It is therefore improbable that the reason for the synteny conservation is the co-expression of these genes.

### Evaluation of *Pdcd2 *as a candidate for *Hst1*

To evaluate the *Pdcd2 *gene as a candidate for the *Hst1*, its testicular transcript was isolated by PCR from the C3H/J mouse testicular library and sequenced. The C3H/J strain carries the *Hst1*^*f *^allele, but it has the same predicted protein product as B6 (*Hst1*^*S *^allele). No polymorphism between B6 and C3H/J strains was detected in 249 bp of the promoter region of *Pdcd2 *(data not shown).

**Figure 9:**
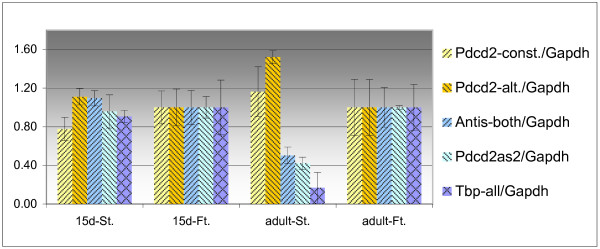
**Relative quantification of the expression of *Pdcd2 *and *Tbp *transcripts in the testes of fertile (Ft.) and sterile (St.) hybrids at the age of 15 days (15d) and adult**. The values were normalized by the expression of *Gapdh *and compared relatively to the expression of the particular transcript in the testes of a fertile hybrid 15 days old and of an adult, respectively.

In 15-day-old mice, significant differences between the frequency of pachytene spermatocytes in the testis of prospectively fertile and sterile hybrids can be detected (10% versus 2% [30]), suggesting that *Hst1 *could be differentially expressed at this stage. The amount of the *Pdcd2 *transcripts was therefore compared in fertile and sterile hybrid mouse testis by Northern blotting with the β-actin probe used as a standard (data not shown) and by QRT-PCR using the *Gapdh *gene as a standard (Fig. 9). The alternative *Pdcd2*, *Pdcd2as1 *and *Pdcd2as2 *were also tested by QRT-PCR. There were no significant differences in the expression of these four transcripts between the prospectively fertile and sterile 15-day-old hybrids. Therefore, *Pdcd2 *is unlikely to be the *Hst1 *gene. A significant upregulation of the *Tbp*, *Pdcd2as1 *and *Pdcd2as2 *transcripts was detected in fertile compared to sterile adult hybrids (Fig. 9). This difference is probably caused by distinct cellular composition of adult fertile and sterile testes that differ in the number of pachytene spermatocytes and spermatids [30]. These cell types vary in the pattern of expression of *Tbp *transcripts (Fig. 6).

### Analysis of genomic imprinting of mouse *Pdcd2 *and neighboring genes

Differential expression of the mouse *Pdcd2 *gene was reported in E9.5 parthenogenetic versus androgenetic mouse embryonic tissues of the B6 strain, suggesting it may be paternally imprinted [21]. Nikaido and colleagues have also suggested that the chromodomain 1 (*Chd1*) gene located 180 kilobases from *Pdcd2 *is maternally imprinted. The imprinting of *Pdcd2 *and neighboring loci could explain the conservation of the synteny. To determine whether the *Pdcd2 *gene and its neighbors are imprinted, polymorphisms in their transcripts were searched comparing the sequences of the mouse strains B6 and PWD/Ph (henceforth PWD) [31]. One single-nucleotide polymorphism (SNP) was detected in the coding region of the constitutive *Pdcd2 *transcript. This synonymous G/A transition is located in exon 5 and thus not present in the alternative *Pdcd2 *transcript (Additional file 2). Primers used to amplify the constitutive mRNA were from exons 2 and 5 and did not detect the *Pdcd2as2 *transcript. The SNP was analyzed in *Pdcd2 *transcripts from RNAs of E9.5 embryos and placentas obtained from reciprocal backcrosses (B6 × (PWD × B6)) and ((B6 × PWD) × B6). Backcross animals were used to control the contamination of placentas by maternal tissues. The DNAs of the embryos were genotyped for Chr 17 and the RNAs from placentas of B6/B6 embryos were tested by RT-PCR for a B6/PWD polymorphic marker in *Tbp *(PWD-specific 6-bp insertion). We found that the placentas were strongly contaminated by maternal tissue and thus were not useful for testing the imprinting status (data not shown). Therefore, the imprinting status of the *Pdcd2 *gene was determined only in the embryos and yolk sacs from reciprocal F1 crosses and backcrosses of B6 and PWD. As both B6 and PWD alleles were present in B6/PWD heterozygous embryos, the *Pdcd2 *constitutive transcript was imprinted neither in mouse E9.5 embryo nor in yolk sac. Polymorphisms were also detected in *Pdcd2as1 *and *Pdcd2as2 *mRNAs in the region not overlapping with the *Pdcd2 *transcripts as well as in five other genes from this region, including *Chd1*, *Tbp*, *Psmb1*, *D17Ph4e*, and *Dll1 *(Additional file 2). All these genes were tested for imprinting in the same way (data not shown). None of these genes was imprinted.

## Discussion

We have analyzed in detail the transcription pattern of the putative apoptotic gene *Pdcd2 *[9] and the tightly linked *Tbp *gene, a key factor in transcription initiation of all eukaryotes [32].

One new alternative *Pdcd2 *transcript, arising by alternative splicing, and at least three new transcripts of *Tbp *originated by alternative polyadenylation have been identified in the mouse. The presence of the alternative *Pdcd2 *mRNA was also found in human [GenBank: NM_144781] and chicken. Although the mechanism and structure of these transcripts is different, in all three species the alternative *Pdcd2 *transcript encodes the MYND zinc-finger domain but not the highly conserved CT domain, suggesting the biological importance of such truncated protein. The function of the alternative *Pdcd2 *product can be deduced from the reported study of cell-cycle regulation [14]. The cell line used has a mutation in the *HCFC1 *gene, causing an arrest of the cell growth at the non-permissive temperature. Transfection of a construct carrying the functional *HCFC1 *gene rescued the arrest. Cotransfection of the complete *PDCD2 *greatly decreased the colony formation, while cotransfection with a truncated *PDCD2*, consisting just of the MYND domain (as in the alternative transcripts), increased it [14]. The function of the alternative *PDCD2 *is therefore likely to be opposite to that of the constitutive form. The ratio of the constitutive and alternative *Pdcd2 *mRNAs differs in the tissues, suggesting a developmental role.

We identified four alternatively polyadenylated alternative forms of *Tbp *in the mouse. The regulation of the stability of *Tbp *mRNAs could be a potential role for all identified alternative forms of *Tbp*, as the 3' UTR determines the life-time of mRNA [34]. Moreover, two of four newly identified transcripts of the *Tbp *gene overlap with *Pdcd2 *transcripts forming the SA pairs. The overlapping transcription of some alternative *Pdcd2 *and *Tbp *transcripts was also confirmed in the chicken. The conservation of SA transcription could explain the conserved synteny of these two genes, although no correlated expression of *Tbp*, *Pdcd2 *and *Psmb1*, three genes of conserved synteny [3], was observed. The mouse *Pdcd2as1 *transcript overlaps just the alternative *Pdcd2 *transcript, while *Pdcd2as2 *overlaps the entire *Pdcd2 *gene (both *Pdcd2 *forms). We determined that their level of transcription represents the minority (about 30% in six mouse tissues, 3% in the testes) of total *Tbp *transcription and that they are mostly localized in the cell nucleus. Both these facts suggest that they play some other regulatory role in gene expression than the protein coding one. Moreover, they are transcribed at a level sufficient to interfere with the transcription of the neighboring *Pdcd2 *gene. A possible mechanism of function of the *Pdcd2as2 *transcript could be the downregulation of *Pdcd2 *expression at the level of transcription via antisense-induced methylation of the CpG island of *Pdcd2 *or by post-transcriptional degradation of mRNA after the formation of dsRNA [35]. Antisense transcripts can participate in the regulation of the ratio between mRNA isoforms [23,35-37]. Correlated expression of the *Pdcd2 *transcripts and overlapping antisense RNAs was suggested by our quantitative expression studies in mouse tissues, but it was not confirmed by the quantification of these transcripts in various developmental stages of the testis nor in flow-sorted populations of spermatogenic cells. The pattern of the expression of *Pdcd2*, *Tbp*, and *Psmb1 *genes in sorted populations of spermatogenic cells is in agreement with the Affymetrix microarray data of Namekawa et al. [38], measuring gene expression in STAPUT-sorted spermatogenic cell populations. Schmidt and Schibler [28,39] found that the *Tbp *gene is upregulated in the testis. We have confirmed their suggestion that the increased transcription of *Tbp *begins already in pachytene spermatocytes (Fig. 6). On the other hand, mRNAs of both the *Pdcd2*-antisense *Tbp *alternative transcripts are present at a higher level in spermatocytes and downregulated in spermatids, suggesting a distinct role for these mRNAs and the constitutive *Tbp *transcript in these cells.

There are several possible reasons why we could not detect any SA relationship. First, the antisense transcript could influence the expression of its sense partner at the level of mRNA translation. This regulation is typical for micro-RNAs and small RNAs, which inhibit the target mRNAs without destroying the template [40,41]. However, this mechanism is improbable, as micro-RNAs do not show perfect complementarity with their target sequence in the majority of eukaryotes [42,43]. To test this hypothesis, the protein level of alternative and constitutive *Pdcd2 *needs to be determined. Second, the antisense regulation could be very dynamic or specific; the rapid change of SA ratio could be detectable just after specific stimuli in particular tissues or cells [42].

We excluded that the antisense transcripts could drive the expression of the sense RNA via mechanisms connected with massive A/I editing of long perfect dsRNA duplexes, resulting from the antisense transcription and causing subsequent retention of the edited transcripts in the nucleus [25,26], as we did not detect any difference in the sequence of cDNA of the alternative *Pdcd2 *exon prepared from the nuclear RNA fraction. Moreover, we did not find any dramatic difference in the nuclear vs. cytoplasmic ratio of *Pdcd2 *transcripts in comparison to control genes. Our results also suggest that the heterogeneity of the cell types in the testes was not the reason of the absence of any SA relationship through the measurement of *Pdcd2*-*Tbp *SA expression in the homogenous testicular cell populations. The proper biological function of *Pdcd2as1 *and *Pdcd2as2 *transcripts thus remains to be determined.

A large number of putative antisense transcripts have been found recently by mapping ESTs and/or cDNAs in the mouse genome [44-47]. However, only a relatively small number of SA loci have been experimentally characterized by expression analysis [42,48]. Here we present the identification and detailed characterization of a specific SA locus. The recent large-scale studies [47,49] suggested that 63 to 72% of all genome-mapped transcription units in the mouse overlap with antisense cDNA or EST and multiple-sized transcripts are often generated from the SA loci [48]. In general, over 65% of transcriptional units produce multiple splice variants, and transcript diversity also arises through alternative promoter usage and alternative polyadenylation [49]. The significance of gene overlap has been shown [50] by an evolutionary approach, as genes overlapping in mammals are more likely to have the same organization in the pufferfish. The *Pdcd2*/*Tbp *SA overlap is conserved in the mouse and chicken, suggesting its biological relevance.

We did not find any support for the candidacy of *Pdcd2 *for *Hst1 *by allelic sequencing and transcription analysis. Our data indicate that *Pdcd2, Chd1*, and four other genes from this region are not imprinted in E9.5 embryos as was suggested by Nikaido et al. An explanation for these conflicting results could be that *Pdcd2 *and *Chd1 *are non-imprinted, but regulated by imprinted genes. Alternatively, the conflict may be due to the use of placentas contaminated by maternal tissues by Nikaido et al. [21]. It has been shown recently that the expression profiling of uniparental mouse embryos is highly inefficient in identifying novel imprinted genes [33].

## Conclusion

The alternative *Pdcd2 *transcripts, which encode the DNA-binding MYND zinc-finger domain but not the CT domain, were identified in the mouse, human and chicken, suggesting the biological importance of such putative truncated protein. The alternative PDCD2 protein lacking the CT domain could interfere with the regulatory effect of the complete PDCD2 protein on gene expression of specific genes. The ratio of the constitutive and alternative *Pdcd2 *mRNAs differs in various tissues, suggesting a developmental role.

The identified *Tbp-*alternative *Pdcd2*-antisense transcripts seem to play some regulatory role in gene expression, compared to the protein-coding function of the constitutive *Tbp *mRNA. They are mostly localized in the cell nucleus and transcribed at a level sufficient to interfere with the transcription of the *Pdcd2 *gene. The conservation of *Pdcd2*/*Tbp *SA overlap in the mouse and chicken also point out their biological relevance. Furthermore, our data suggest that at least some of the cDNAs identified in the large-scale sequencing projects labeled as noncoding RNAs are in fact incomplete alternative cDNAs of neighboring protein-coding genes.

## Methods

### RNA preparation, reverse transcription, real-time PCR

Total RNA was prepared using Trizol (Invitrogen). Reverse transcription (RT) was performed with MuMLV or SuperScript II (Invitrogen). Quantification of mRNAs was performed using a real time PCR system (LightCycler, Roche). An aliquot corresponding to 50 ng of total RNA was added to the reactions with FastStart DNA Master SYBR Green I kit and cycled in the LightCycler. As controls, RT reactions without reverse transcriptase were utilized. The assays were done several times with independently collected samples. The data were analyzed using LightCycler Software version 3.5.3 (Roche).

### Polymerase chain reaction (PCR) and sequencing

Each template or control was added to 50 μl reactions prepared as master mixes containing final concentration 0.15 nM of each of the four dNTPs, 50 mM KCl, 10 mM Tris-HCl, pH 8.8, 1.6 mM MgCl_2_, 0.08% Nonindet P40, 1.5 *U *of Taq polymerase (MBI Fermentas), and 15 pmol of each primer (see Additional file 1 for all used primers). Additives betaine (1 M, Sigma) and/or DMSO (7%, Sigma) were used for GC-rich templates. The amplification products were denatured in a thermal cycler (Applied Biosystems) at 94°C for 2 min, then cycled 25 to 38 times at 94°C for 30 s, at the corresponding primer annealing temperature for 30 s, and at 68°C for 2.5 min, and, finally, incubated at 70°C for 5 min. The PCR products were resolved on agarose gels with ethidium bromide along with a size marker and photographed under UV illumination to check their sizes. The sequencing reactions were performed with the Big Dye kit according to the manufacturer's instructions (Applied Biosystems) and run in a sequencing capillary machine ABI310 (Applied Biosystems).

### Isolation of the 3' end of *Pdcd2 *alternative and antisense transcript by 3' RACE

3' RACE experiments were performed with 0.5 μg of total 28-day-old mouse testes RNA and 10 pmol of the poly-T primer 3'CDS according to the manufacturer's instructions (SMART RACE Kit, Clontech). The first-strand cDNA synthesis was performed at 50°C for 30 min in 540 mM Trehalose, 1× RACE first strand buffer (Clontech), 10 mM DTT, 1 mM dNTPs, and 100 units SuperScript reverse transcriptase (Invitrogen). After incubation, first-strand cDNA reaction was diluted 1:5 with water and incubated at 75°C for 10 min to inactivate the reverse transcriptase.

Two-and-half microliters were used for PCR reactions in a total reaction volume of 50 μl and subjected to 35 cycles of amplification. Each cycle consisted of a 94°C denaturation (30 s), 56°C annealing (30 s), and 68°C extension (3 min) step. The first round of PCR was performed with 0.5 μM of the gene-specific primer and the Universal primer mix (UPM-Clontech). Reactions with only one primer added served as negative controls.

Ten percent of the PCR reactions were analyzed on agarose gels. PCR products were diluted 1:20 and 1 μl served as a template to a second round of PCR using 0.5 μM nested gene-specific primer, and Nested Universal Primer (NUP, Clontech). Amplifications were performed for 20 cycles using standard PCR conditions. PCR products were visualized on agarose gels, isolated from the gel and sequenced.

### 5'RACE Invitrogen GeneRacer kit

The 5' end of the *Updcd2 *transcript was cloned and identified with GeneRacer kit (Invitrogen) using gene-specific *Updcd2 *Racer primers (Additional file 1). This method is designed to capture only the intact full-length transcripts while eliminating any truncated mRNA. Total RNA from mouse testes was first treated with calf intestinal phosphatase to dephosphorylate the 5'-ends of any truncated mRNA. Following decapping the intact mRNAs with tobacco acid pyrophosphatase, GeneRacer RNA oligo was ligated specifically to the 5'-ends of the decapped mRNAs. After the synthesis of the first strand cDNA using the oligo- [dT] primer with Superscript II RNase H- reverse transcriptase, the 5'-end of the *Pdcd2 *mRNA was amplified by PCR followed by nested PCR according to the procedure described in the GeneRacer kit using *Pdcd2 *gene-specific primers, and the 5' primer and nested 5' primer supplied by the kit. The PCR products were isolated, cloned and sequenced.

### GenBank accession numbers

DQ906042- *Mus musculus Pdcd2 *alternative transcript mRNA, complete cds,

DQ906043- *Mus musculus Pdcd2*-antisense 2 (*Pdcd2as2*) transcript, *Tbp *mRNA with alternative 3'UTR, partial cds,

DQ906044- *Mus musculus *upstream *Pdcd2 *(*Updcd2*) noncoding mRNAs sharing the *Pdcd2 *promoter, partial sequence,

DQ906045- *Gallus gallus *PDCD2 alternative transcript mRNA, complete cds,

DQ906046- *Gallus gallus *PDCD2 transcript with alternative 3'UTR (a3PDCD2) mRNA, partial sequence,

DQ906047- *Gallus gallus *TBP alternative transcript 2 (cTBP2), TBP mRNA with alternative 3'UTR, partial sequence.

### Preparation of testicular single-cell suspension and FACS

Cells were isolated from B6 two-month-old male mice by using two-step enzymatic digestion to remove interstitial cells [51], using the EKRB medium (pH 7.2) supplemented with collagenase (50 mg/ml) and DNase (1 mg/ml). The single-cell suspensions were pelleted and resuspended in appropriate buffer.

Two million cells were diluted in 1.5 ml EKRB + 1% fetal calf serum (FCS) with DNase (1 mg/ml) and stained with Hoechst (1 mg/ml) for 1 h at 32°C. Just before analysis, PI (propididium iodide, 1 mg/ml) was added to exclude dead cells. Analysis and cell sorting were performed in FACS (Fluorescence Activated Cell Sorter) Vantage (Becton, Dickinson and Company). One hundred thousands of leptotene-zygotene spermatocytes, pachytene spermatocytes and spermatids for RNA isolation were sorted directly to RLT solution with mercaptoethanol (QIAGEN RNeasy micro kit). Twenty thousand cells of each fraction were sorted to EKRB medium with 1% FCS and Hoechst (1 mg/ml) to check the purity of the spermatocyte fractions after the immunostaining using an antibody against the synaptonemal complex.

### Isolation of the cytoplasmic/nuclear RNA fraction

The testicular single-cell suspensions (5 × 10^7 ^cells) were pelleted and resuspended in 300 μl RLN buffer (50 mM Tris-Cl, pH 8.0, 140 mM NaCl, 1.5 mM MgCl_2_, 0.5% NP-40) supplemented with 1 mM DTT and RNAsin (1000 U/ml). The mixture was incubated on ice for 5 min and spun in a microcentrifuge at 14 000 rpm for 5 min. The supernatant containing the cytoplasmic fraction was separated from the pellet (nuclear fraction). The RNAs were prepared using Trizol (Invitrogen).

## Authors' contributions

OM carried out experimental work described in the paper, participated in designing the study and drafted the manuscript. ZT conceived the study, participated in its design, and together with JF and OM participated in the writing of the manuscript. All authors read and approved the final manuscript.

## Supplementary Material

Additional file 1 **Primers used for RT-PCR, RACE and real-time PCR**. The primer name, 5'-> 3' sequence and usage are noted. In QRT-PCR primer pairs, conditions of the reaction using the real-time PCR system (LightCycler, Roche) are noted in the brackets. Primer names beginning with "Ch" amplify chicken *Pdcd2*, *Tbp *genes. F, forward primer; R, reverse primer; alt., alternative. Click here for file

Additional file 2 **Testing the imprinting status of the transcripts in the *Hst1 *region**. B6 × PWD polymorphisms used to test the imprinting status of the transcripts in the *Hst1 *region are noted in the table. The example of the imprinting analysis (for *Pdcd2 *constitutive transcript) are depicted below. Click here for file

## Figures and Tables

**Table 1:** Absolute quantification of the expression of *Pdcd2 *and *Tbp *transcripts in seven mouse tissues. The values represent the number of copies of the particular transcript in the sample corresponding to the amount of 5 pg of total RNA.

Tissue/Transcript	*Pdcd2*-const.	*Pdcd2*-alt.	*Pdcd2*-both	Antis-both	*Pdcd2as2*	*Tbp*-all
Kidney	134	2	169	70	28	342
Heart	93	2	110	59	14	218
Liver	97	2	104	32	9	136
Brain	80	4	108	171	16	382
Lung	95	3	117	125	24	362
Spleen	158	7	227	171	63	448
Testes	158	4	203	251	36	8140

const., constitutive transcript; alt., alternative transcript
