# Supplementary material for: Conserved alternative and antisense transcripts at the programmed cell death 2 locus
Source: BMC Genomics. 2007 Jan 18;8:20. doi: 10.1186/1471-2164-8-20 (PMC1800895; doi:10.1186/1471-2164-8-20)
Supplement: Additional file 2 — Testing the imprinting status of the transcripts in the Hst1 region. B6 × PWD polymorphisms used to test the imprinting status of the transcripts in the Hst1 region are noted in the table. The example of the imprinting analysis (for Pdcd2 constitutive transcript) are depicted below. [file 1471-2164-8-20-S2.doc]

| **transcript** | **B6 sequence** | **PWD sequence** |
| --- | --- | --- |
| ***Pdcd2*-constitutive** | ATGGAAGAGGGATTAAACCCA | ATGGAAGAGGAATTAAACCCA |
| Pdcd2as1+2 | TTTTCAAAGT-CAGTGCTGAA | TTTTCAAAGTTCAGTGCTGAA |
| Pdcd2as2 | ATCTCAGAAC--AGAGAGTTTC | ATCTCAGAACAGAGAGAGTTTC |
| Psmb1 | TGCAGAATGTAGAGCACGTCC | TGCAGAATGTGGAGCACGTCC |
| D17Ph4e | TCTGCCCTCCGGTCTCAGCAA | TCTGCCCTCCCGTCTCAGCAA |
| Dll1 | ACGGCAGTGCTGTCACGCCAG | ACGGCAGTGCCGTCACGCCAG |
| Chd1 | AAAAGTCTGTAGTGTCCGATG | AAAAGTCTGTGGTGTCCGATG |
| Tbp | AGACAGCAG------CAGCAACAGCAACAG | AGACAGCAGCAGCAGCAGCAACAGCAACAG |

**Example of the imprinting analysis** (constitutive *Pdcd2* transcript). RNAs were isolated from E9.5 embryos obtained from F1 reciprocal hybrids B6 x PWD and PWD x B6. As both B6 and PWD alleles were present in B6/PWD heterozygous embryos, the *Pdcd2* constitutive transcript was not imprinted in mouse E9.5 embryos.

**B6xPWD**


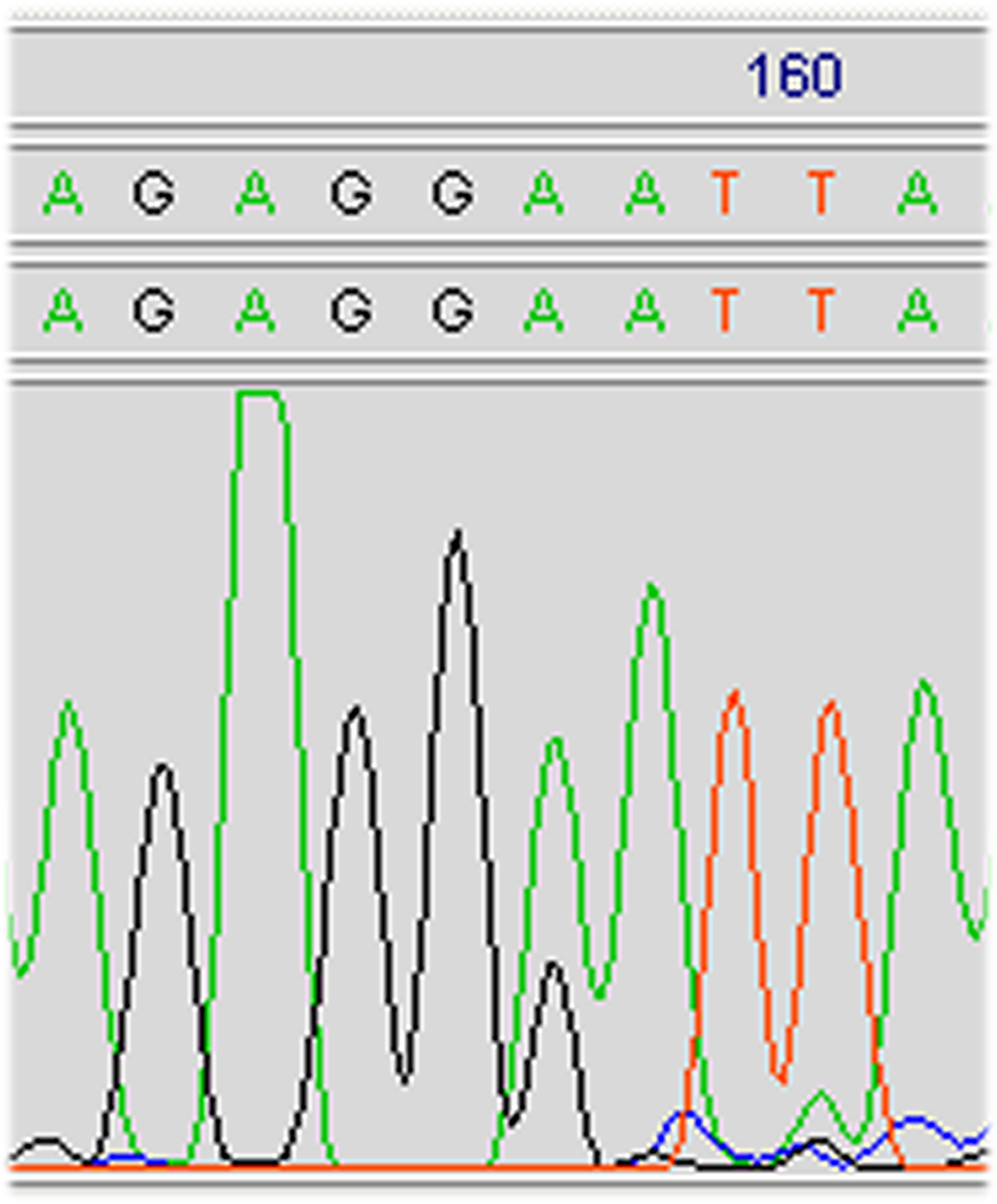

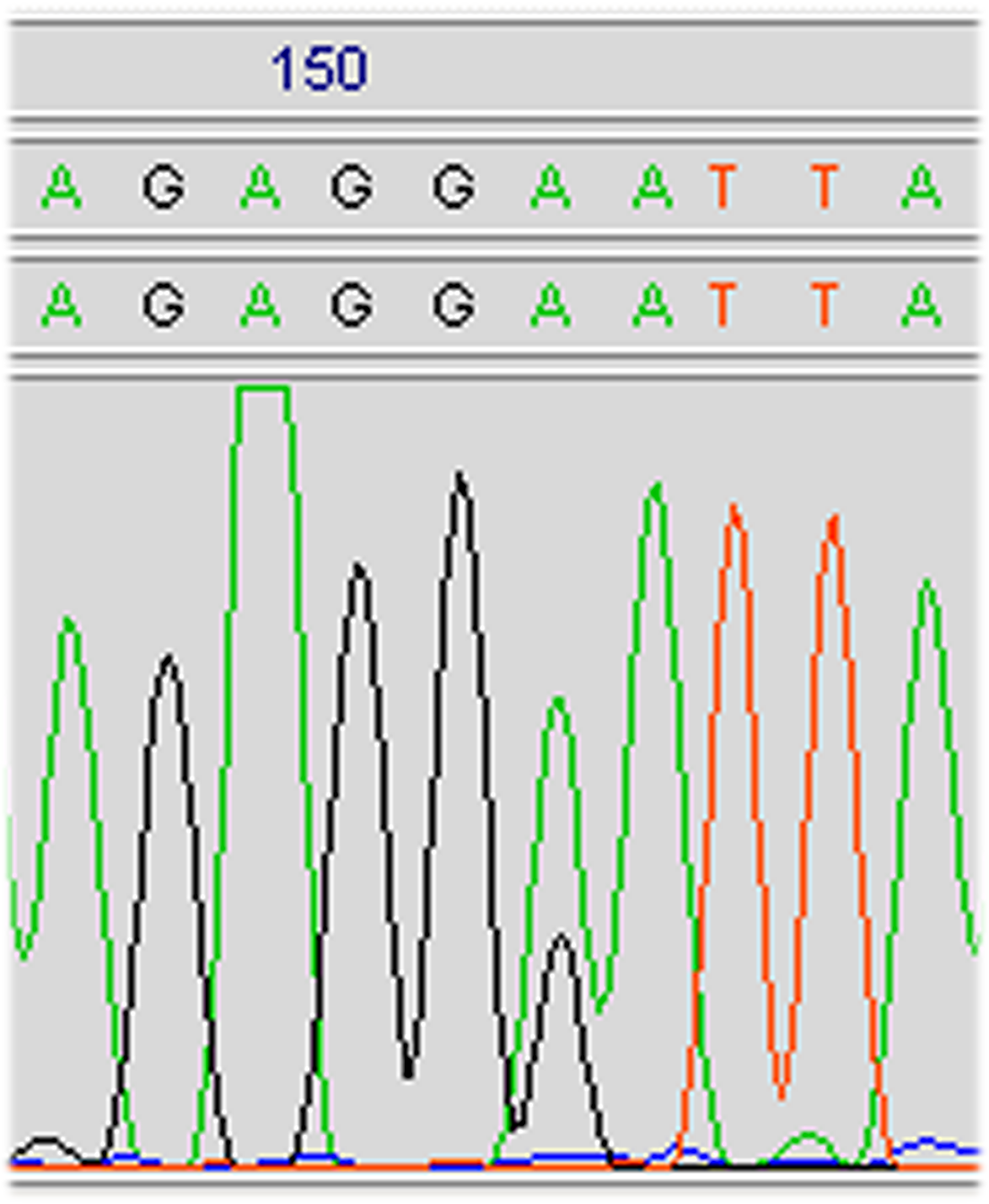


**PWDxB6**
